# Supplementary material for: Noble Metal Complexes of a Bis-Caffeine Containing NHC Ligand
Source: Molecules. 2022 Jul 5;27(13):4316. doi: 10.3390/molecules27134316 (PMC9268361; doi:10.3390/molecules27134316)
Supplement: Supplementary file 1 [file molecules-27-04316-s001.zip › molecules-1760151-supplementary.pdf]

Supplementary Materials

# Noble Metal Complexes of a Bis-Caffeine Containing NHC Ligand

Oliver Bysewski [1,2], Andreas Winter [1,2], Phil Liebing [3] and Ulrich S. Schubert [1,2,*]

[1] Laboratory of Organic and Macromolecular Chemistry (IOMC), Friedrich Schiller University Jena, Humboldtstrasse 10, 07743 Jena, Germany

[2] Center for Energy and Environmental Chemistry Jena (CEEC Jena), Philosophenweg 7a, 07743 Jena, Germany

[3] Institute for Inorganic and Analytical Chemistry (IAAC), Friedrich Schiller University Jena, Humboldtstr. 8, 07743 Jena, Germany

KEYWORDS caffeine, mesoionic carbene, palladium, platinum, NHC

Contents

Figure S1: ESI-MS spectrum of **3**. ........ 2
Figure S2: ESI-MS spectrum of **4**. ........ 2
Figure S3: ESI-MS spectrum of **5**. ........ 3
Figure S4: ESI-MS spectrum of **6**. ........ 3
Figure S5: ESI-MS spectrum of **7**. ........ 4
Figure S6: $^{1}$H-NMR (300 MHz, $CDCl_3$) spectrum of **3**. Asterisk show residual DMF. ........ 5
Figure S7: $^{13}$C-NMR (75 MHz, $CDCl_3$) spectrum of **3**. ........ 5
Figure S8: $^{1}$H-NMR (300 MHz, $CH_3CN$) spectrum of **4**. ........ 6
Figure S9: $^{13}$C-NMR (75 MHz, $CH_3CN$) spectrum of **4**. ........ 6
Figure S10: $^{1}$H-NMR (300 MHz, $CH_3CN$) spectrum **5**........ 7
Figure S11: $^{13}$C-NMR (75 MHz, $CH_3CN$) spectrum **5**........ 7
Figure S12: $^{1}$H-NMR (300 MHz, $CH_3CN$) spectrum of **6**. ........ 8
Figure S13: $^{13}$C-NMR (75 MHz, $CH_3CN$) spectrum of **6**........ 8
Figure S 14: $^{1}$H-NMR (300 MHz, $CH_3CN$) spectrum of **7**. Asterisk show residual DMF........ 9
Figure S15: $^{13}$C-NMR (75 MHz, $CH_3CN$) spectrum of **7**. ........ 9
Figure S16: $^{1}$H-NMR (300 MHz, $CH_3CN$) of the nickel complex. ........ 10
Figure S17: $^{1}$H-NMR (300 MHz, $CH_3CN$) of the ruthenium reaction........ 10
Figure S18: $^{1}$H-NMR (300 MHz, $CH_3CN$) of the iron(II) NHC complex........ 11
Figure S19: Variable temperature $^{1}$H-NMR (300 MHz, DMSO) of **6** from 25 °C (1) to 100 °C (8).... 11
Figure S20: Variable temperature $^{1}$H-NMR (300 MHz, DMSO) of **7** from 25 °C (1) to 100 °C (8).... 12

Table S1: Crystal data and details on structure refinement for **7** · $CH_3CN$........ 13
Table S2: Fractional Atomic Coordinates (×$10^4$) and Equivalent Isotropic Displacement Parameters (Å$^2$×$10^3$) for **7** · $CH_3CN$. $U_{eq}$ is defined as 1/3 of the trace of the orthogonalised $U_{ij}$ tensor. ........ 14
Table S3: Anisotropic Displacement Parameters (Å$^2$×$10^3$) for **7** · $CH_3CN$. ........ 15
Table S4: Bond lengths for **7** · $CH_3CN$. ........ 16
Table S5: Bond Angles for **7** · $CH_3CN$. ........ 17
Table S6: Hydrogen Atom Coordinates (Å×$10^4$) and Isotropic Displacement Parameters (Å$^2$×$10^3$) for **7** · $CH_3CN$. ........ 18

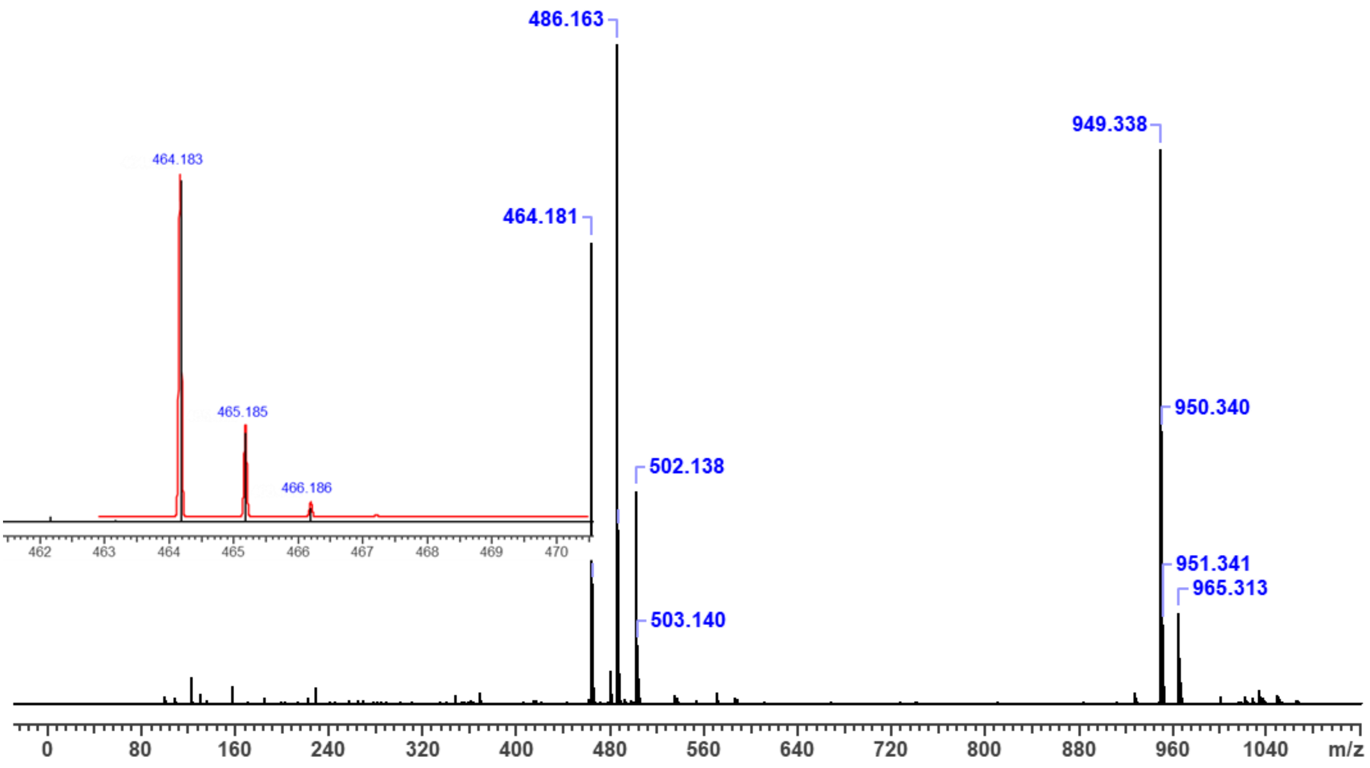


Figure S1: ESI-MS spectrum of **3**.

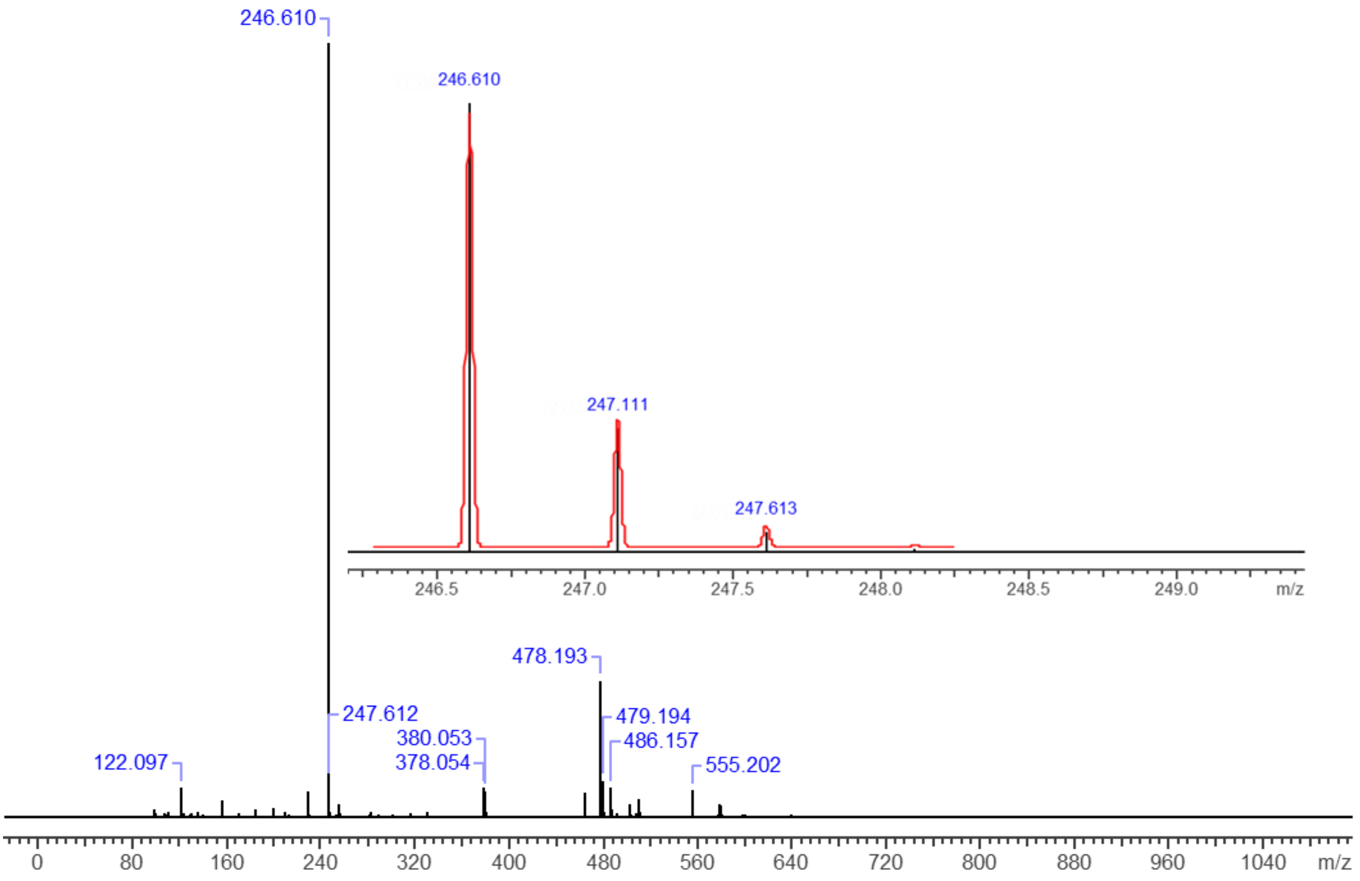


Figure S2: ESI-MS spectrum of **4**.

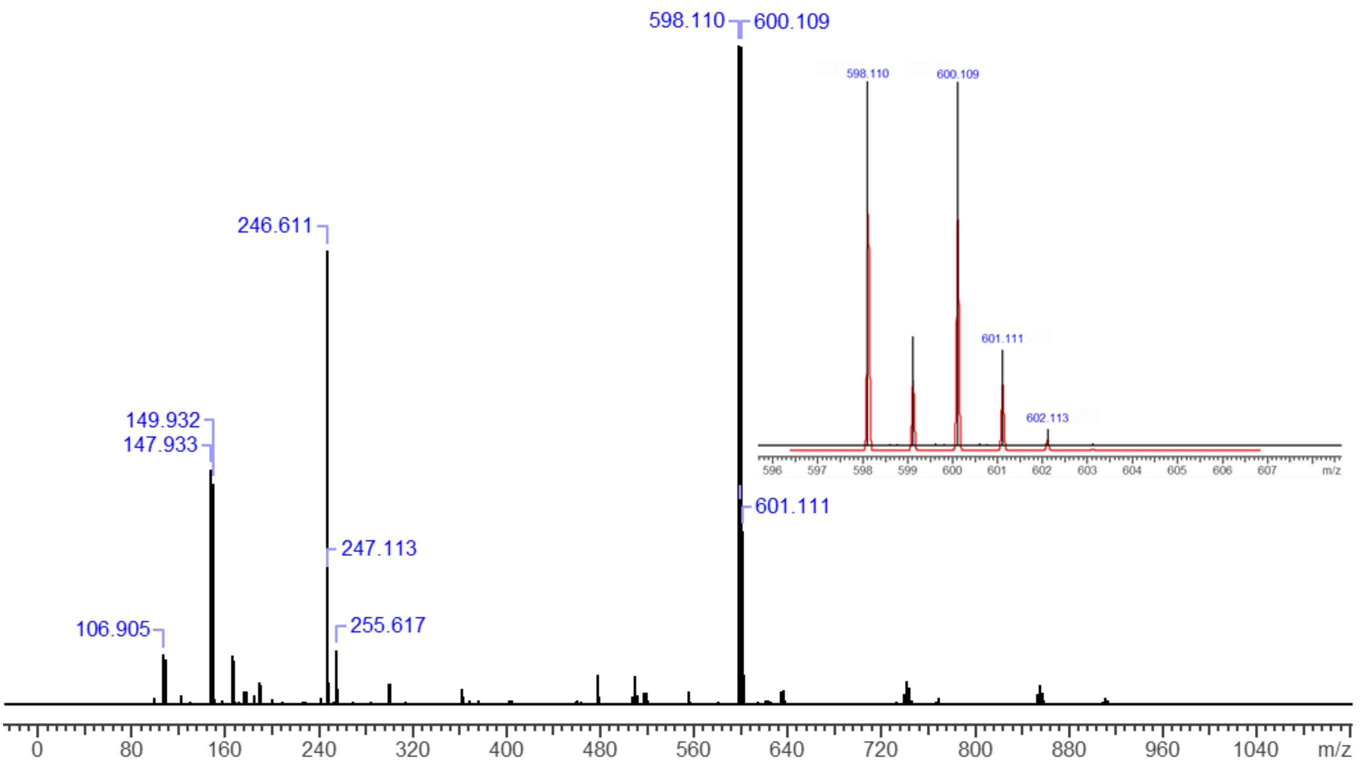


Figure S3: ESI-MS spectrum of **5**.

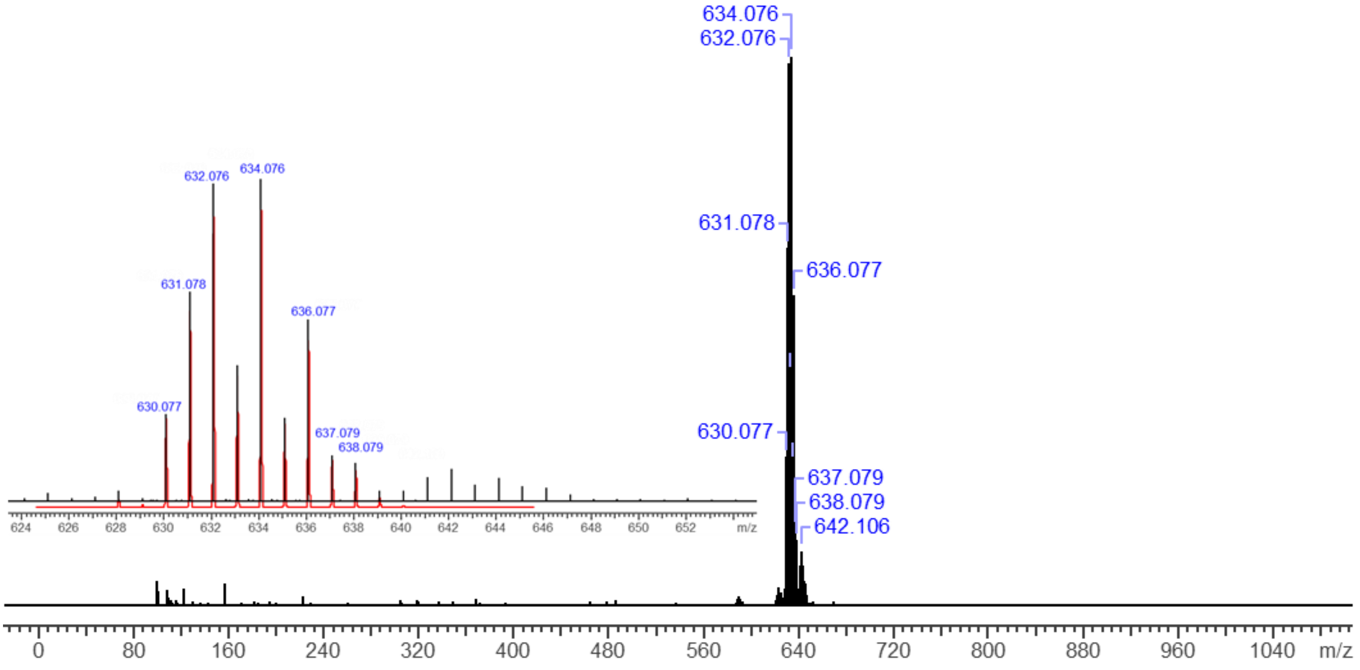


Figure S4: ESI-MS spectrum of **6**.

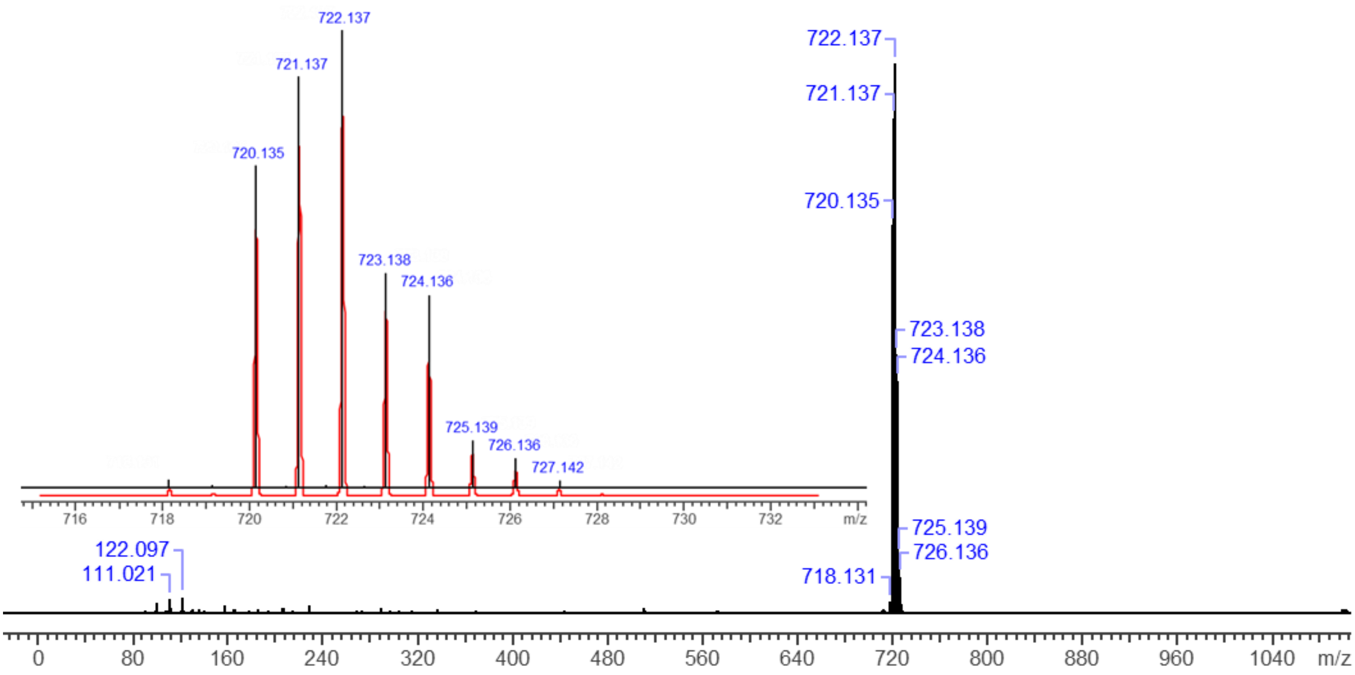


Figure S5: ESI-MS spectrum of **7**.

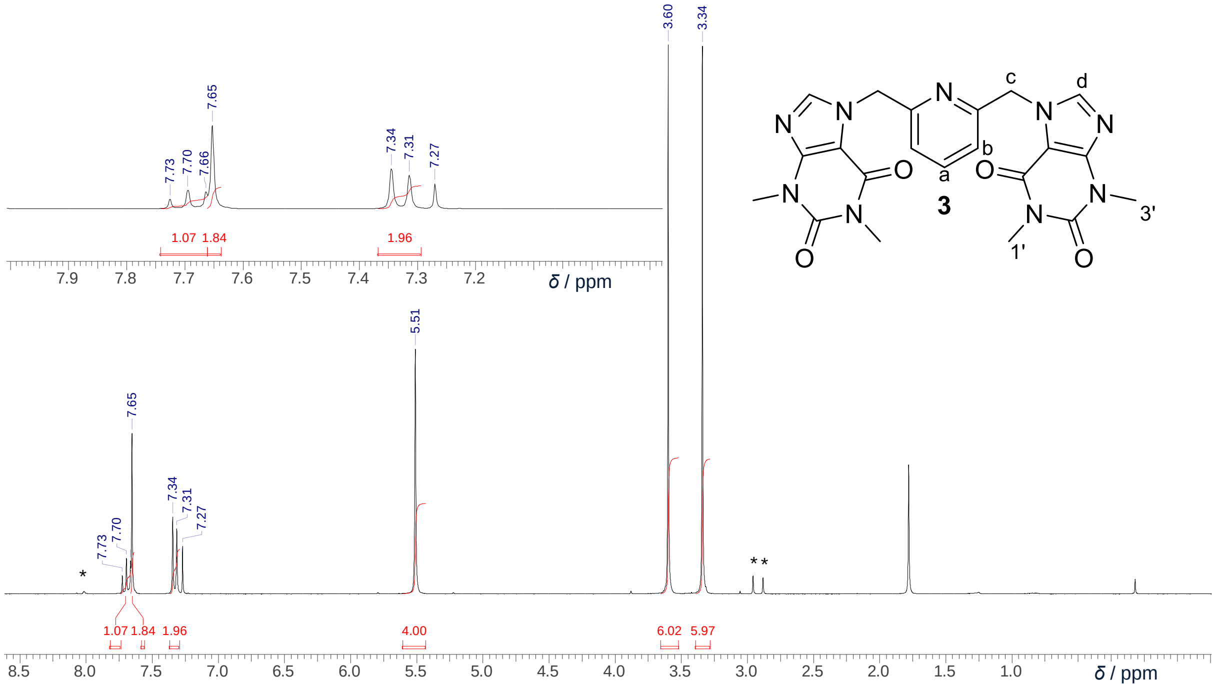


Figure S6: $^{1}$H-NMR (300 MHz, $CDCl_3$) spectrum of **3**. Asterisk show residual DMF.

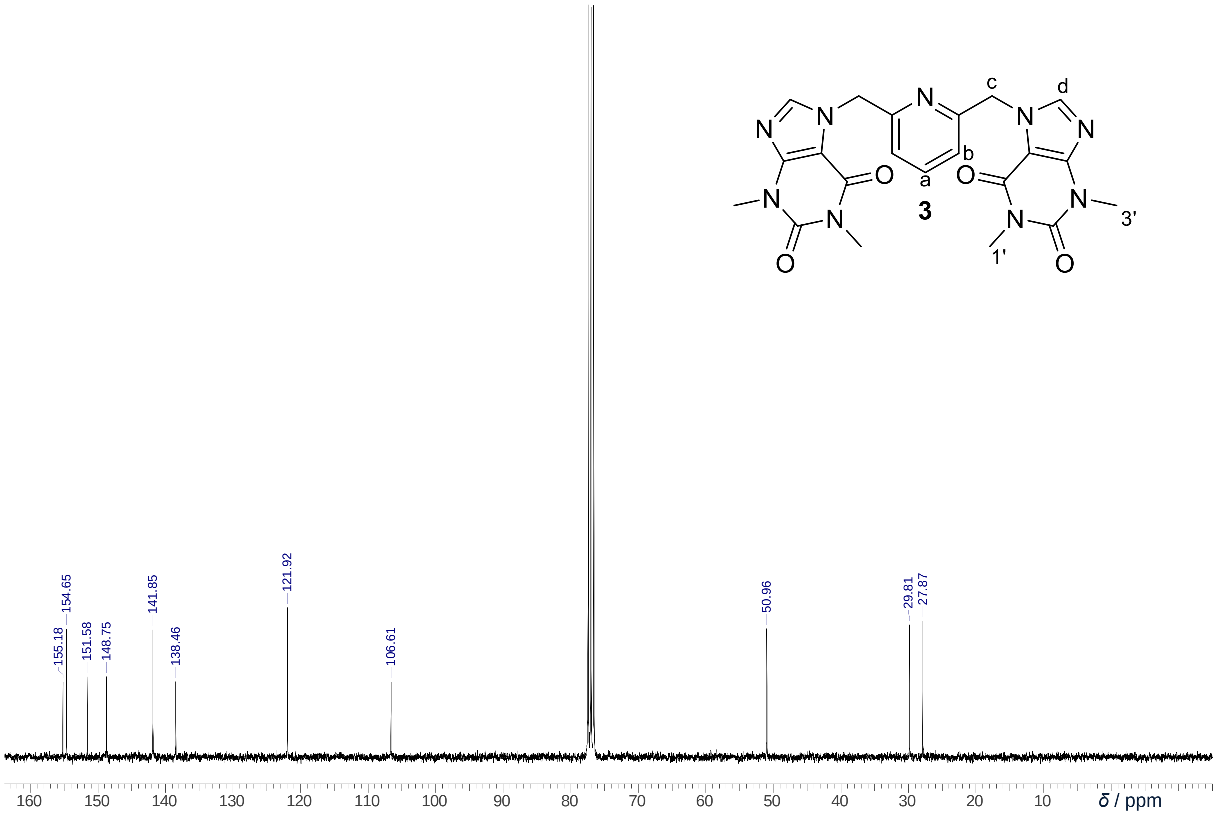


Figure S7: $^{13}$C-NMR (75 MHz, $CDCl_3$) spectrum of **3**.

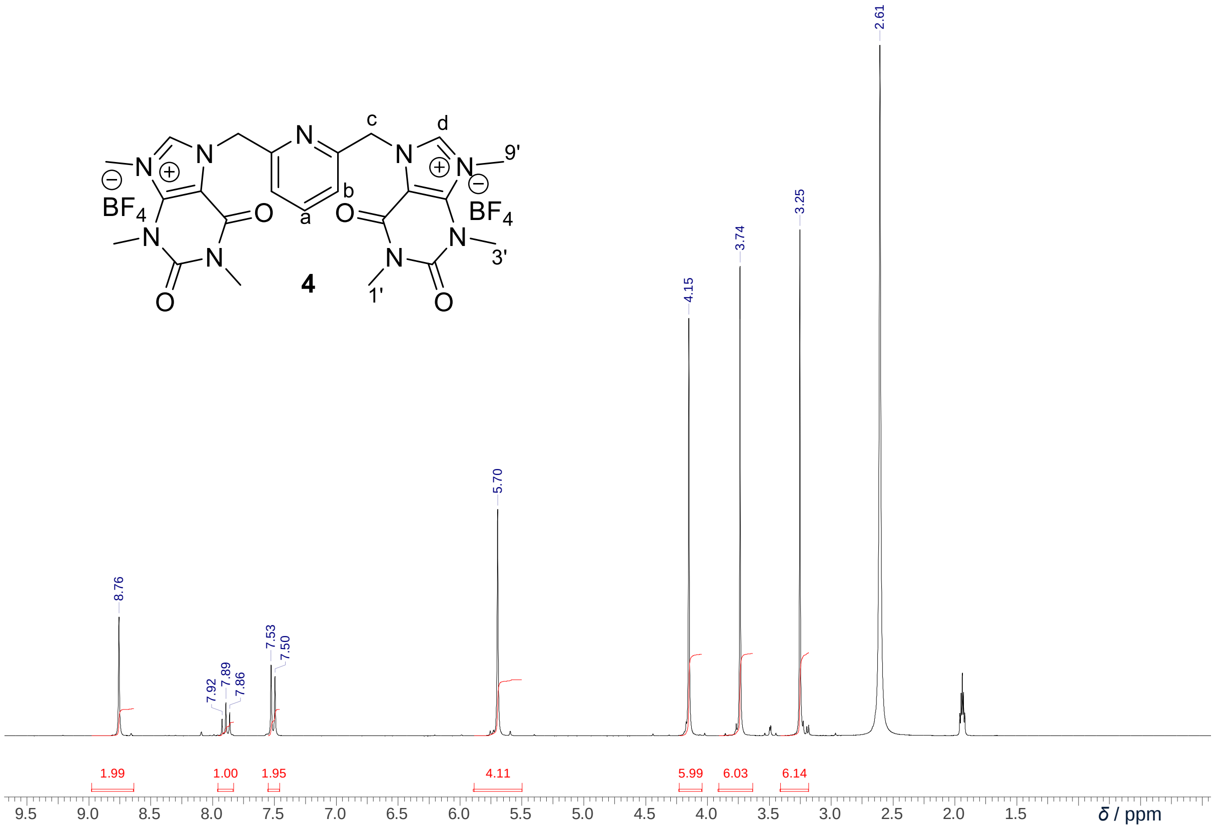


Figure S8: $^{1}$H-NMR (300 MHz, $CH_3CN$) spectrum of **4**.

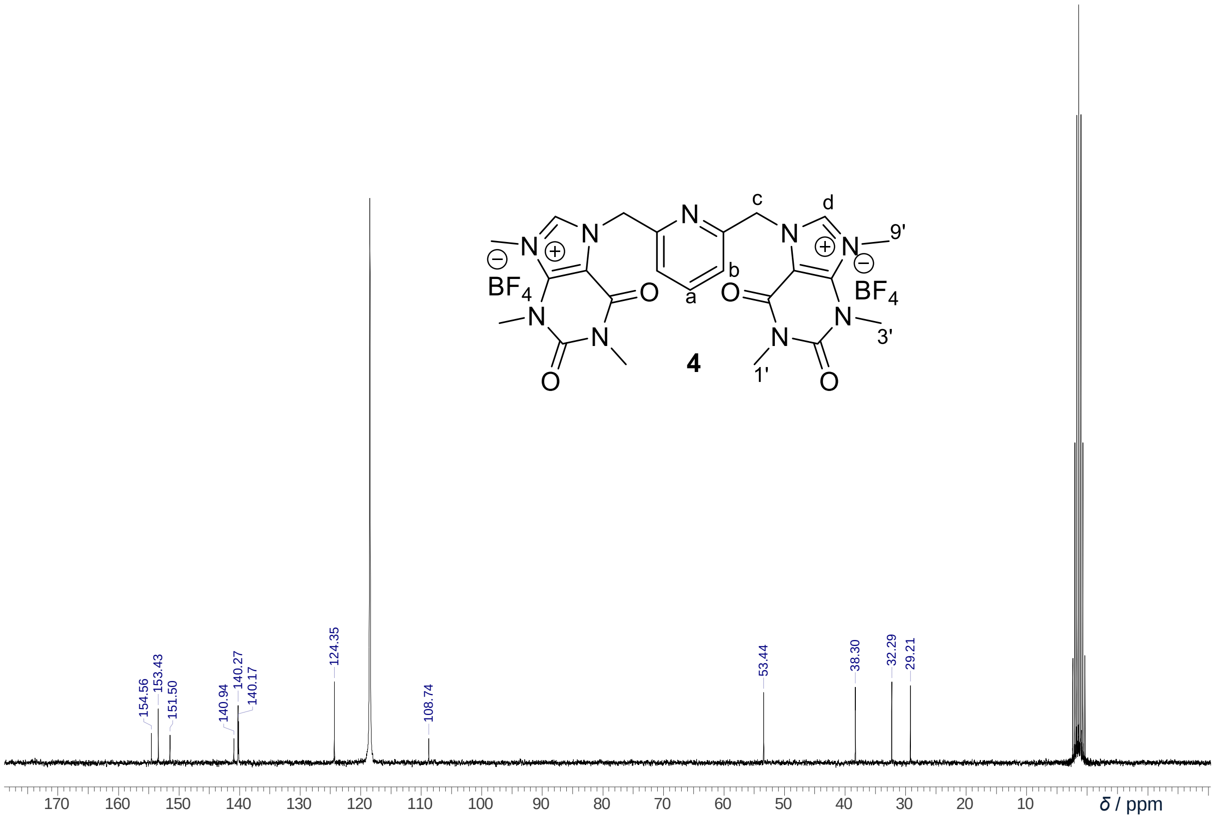


Figure S9: $^{13}$C-NMR (75 MHz, $CH_3CN$) spectrum of **4**.

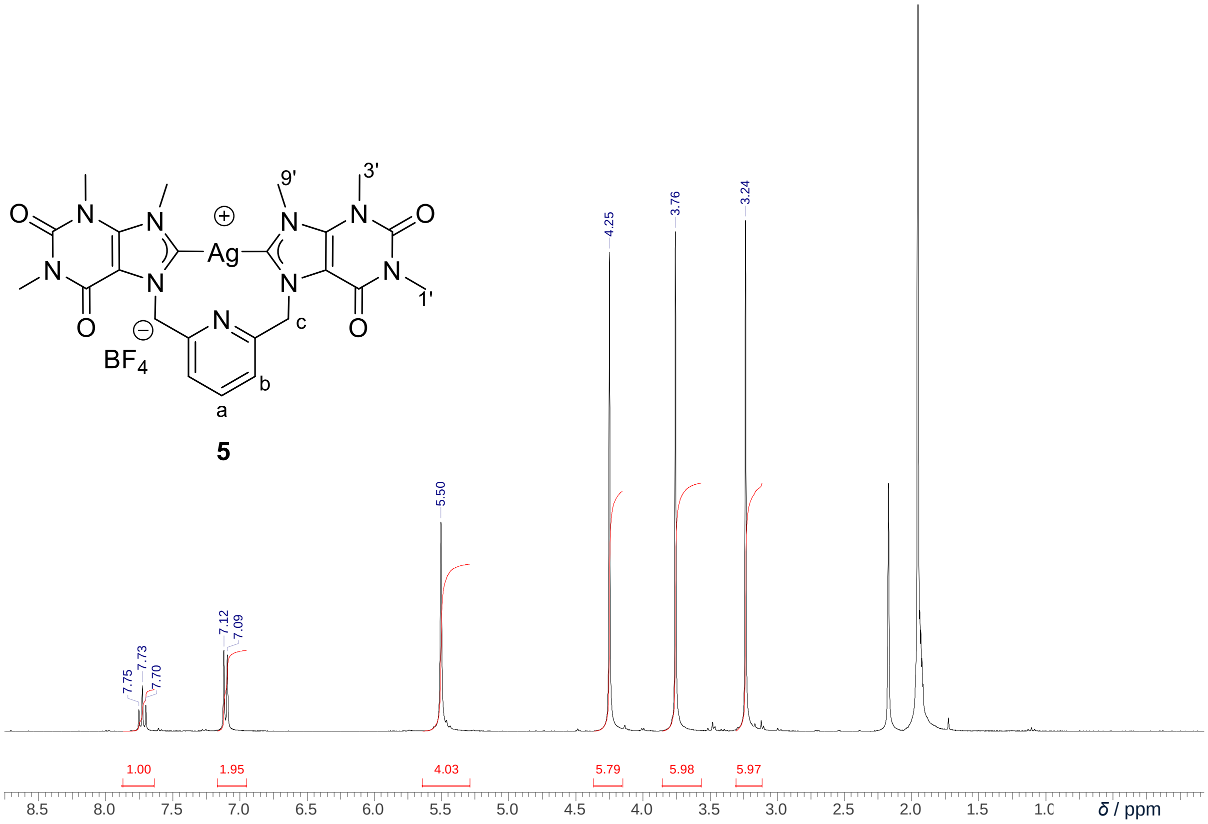


Figure S10: $^{1}$H-NMR (300 MHz, $CH_3CN$) spectrum **5**.

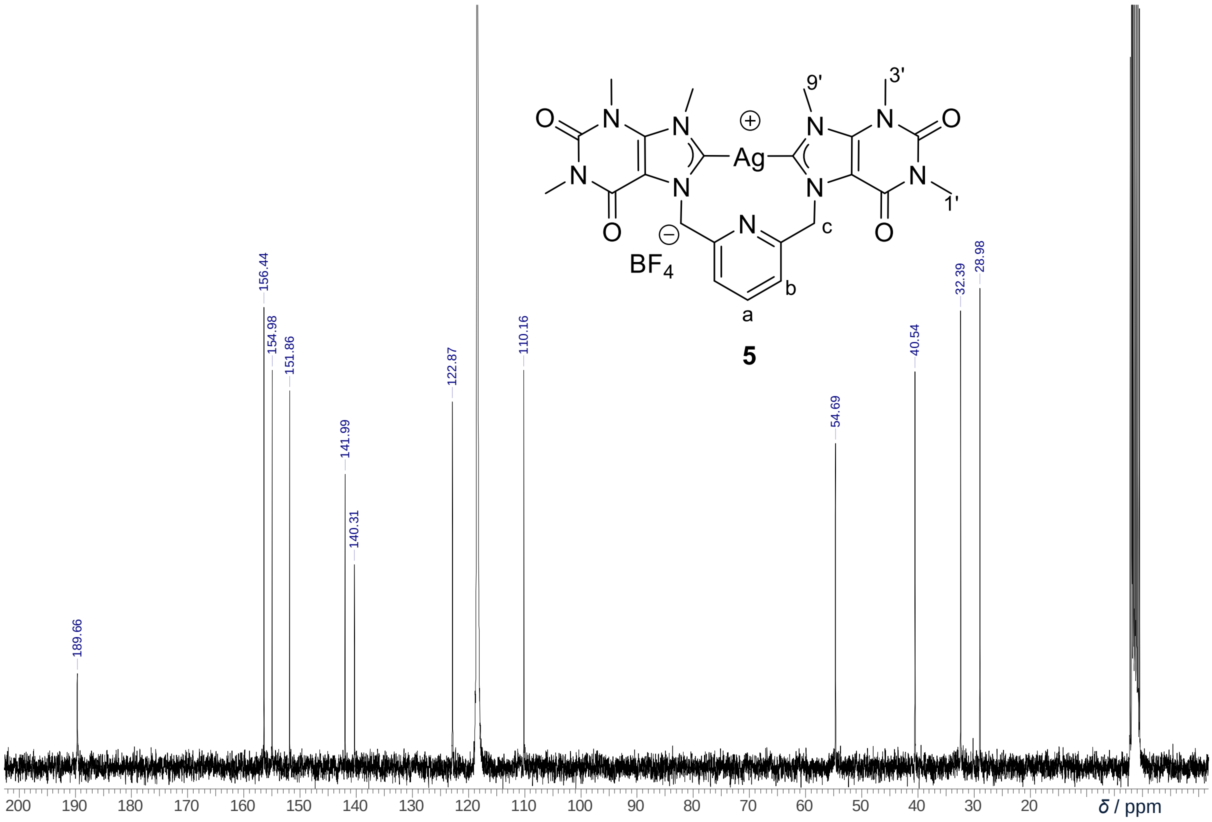


Figure S11: $^{13}$C-NMR (75 MHz, $CH_3CN$) spectrum **5**.

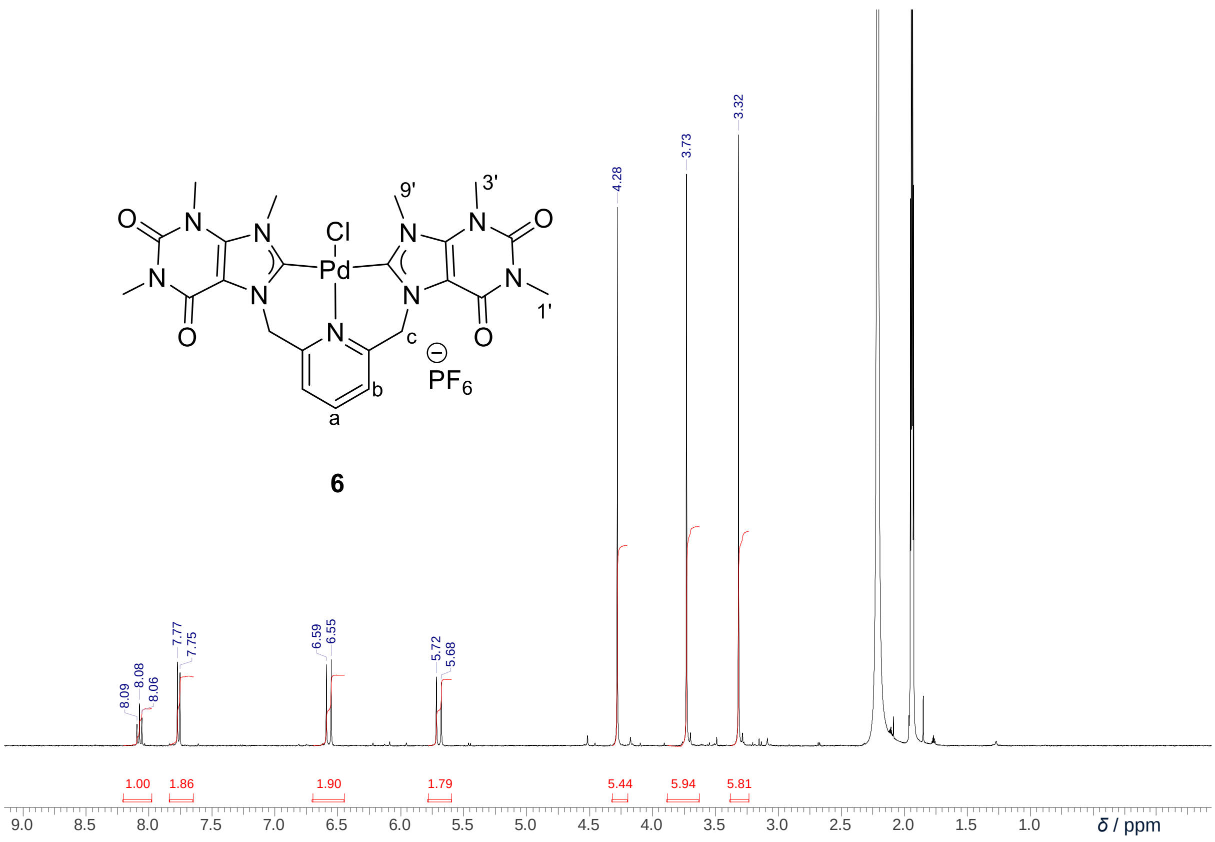


Figure S12: $^{1}$H-NMR (300 MHz, $CH_3CN$) spectrum of **6**.

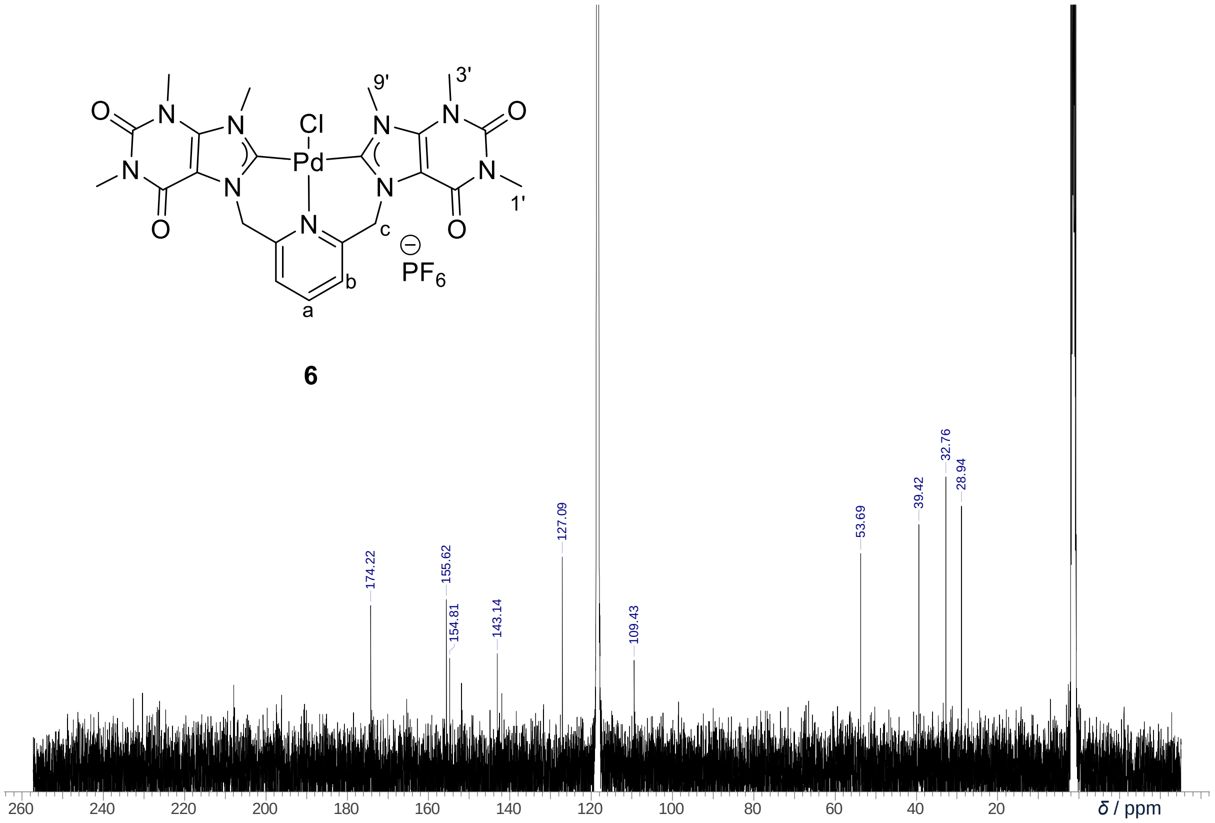


Figure S13: $^{13}$C-NMR (75 MHz, $CH_3CN$) spectrum of **6**.

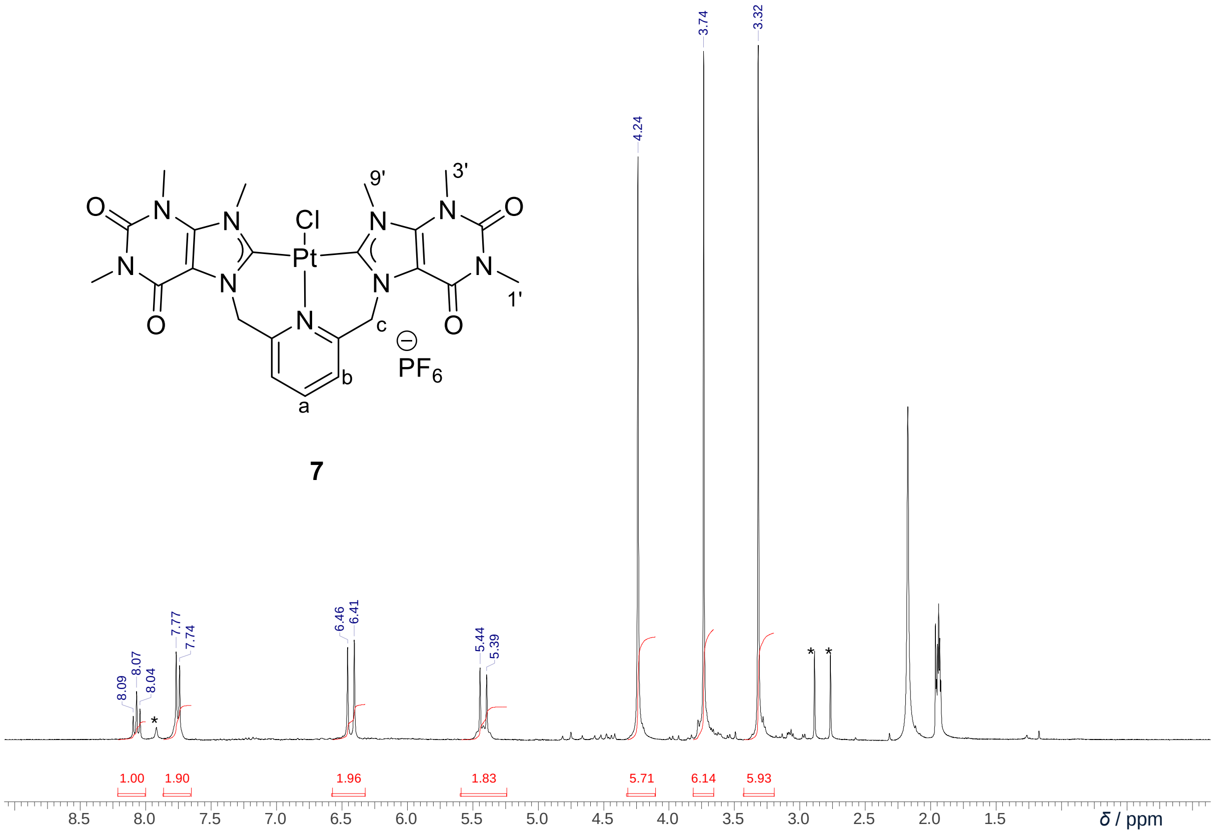


Figure S 14: $^{1}$H-NMR (300 MHz, $CH_3CN$) spectrum of **7**. Asterisk show residual DMF.

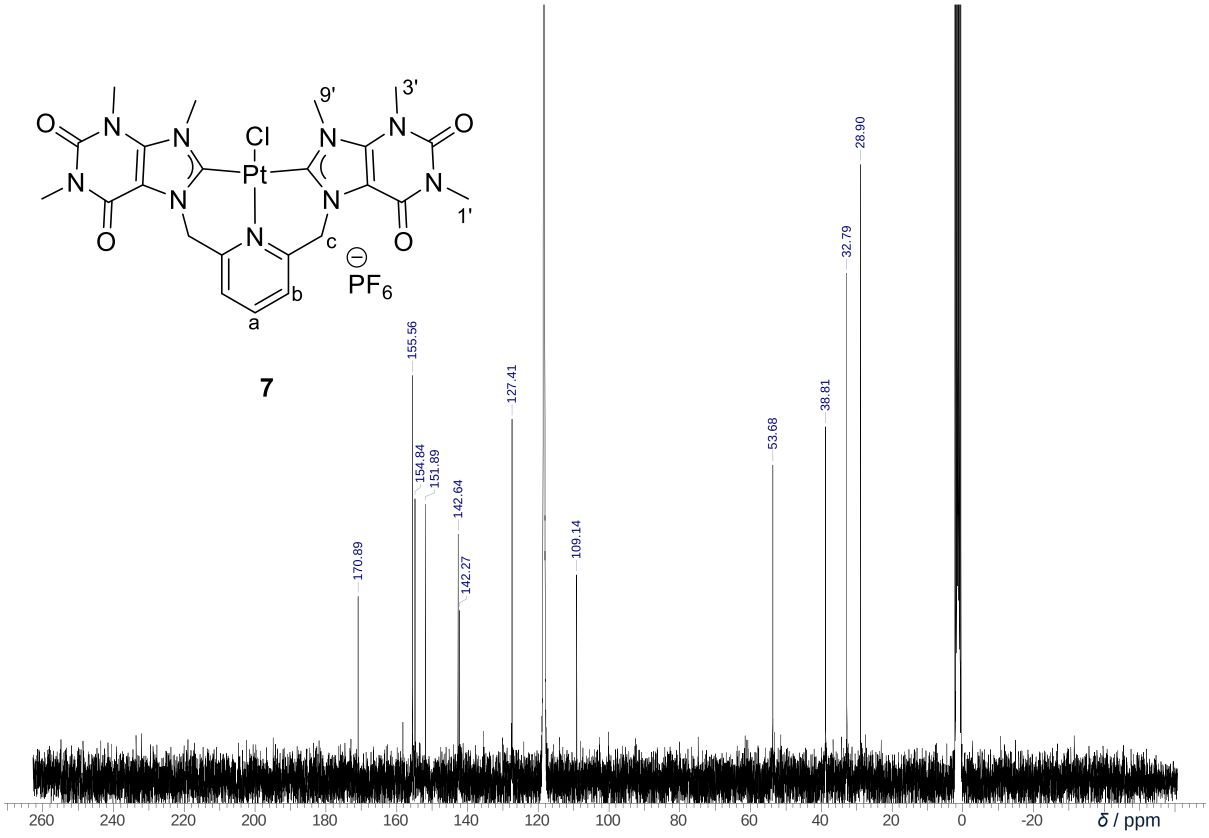


Figure S15: $^{13}$C-NMR (75 MHz, $CH_3CN$) spectrum of **7**.

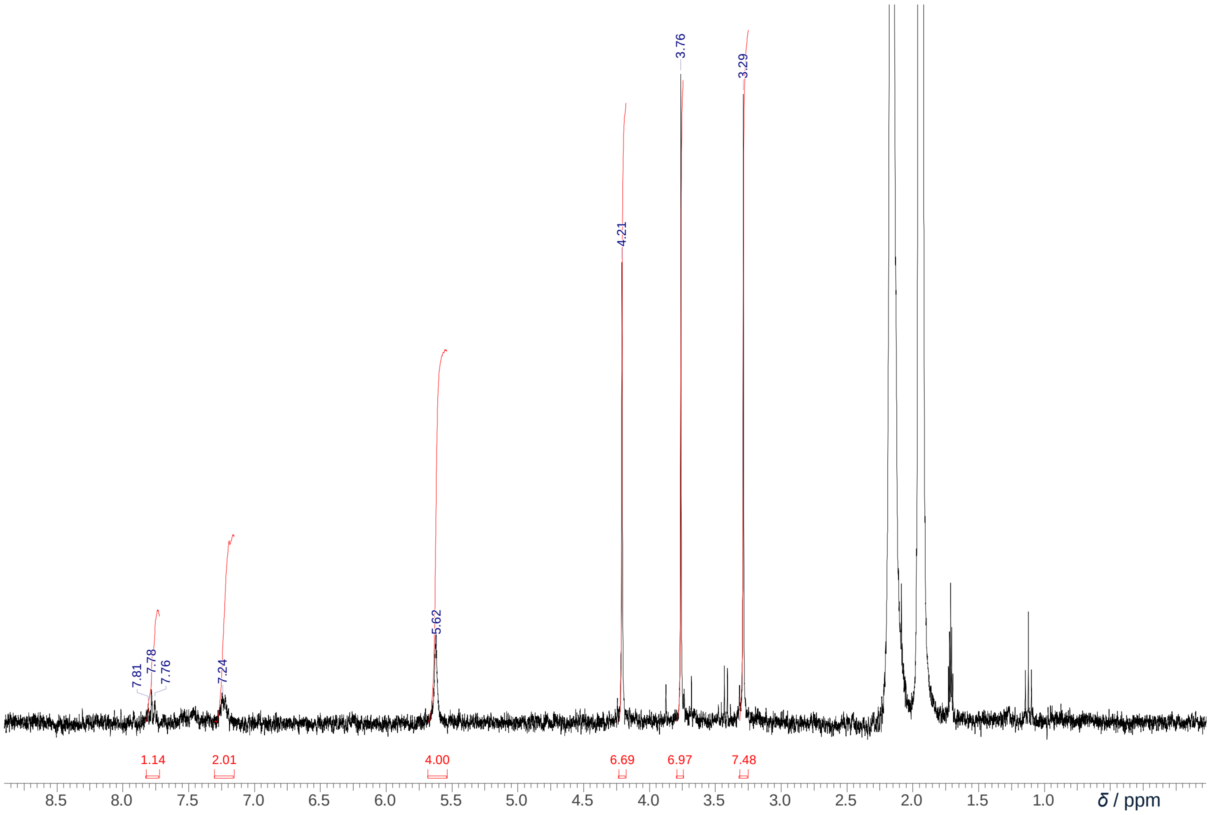


Figure S16: $^{1}$H-NMR (300 MHz, $CH_3CN$) of the nickel complex.

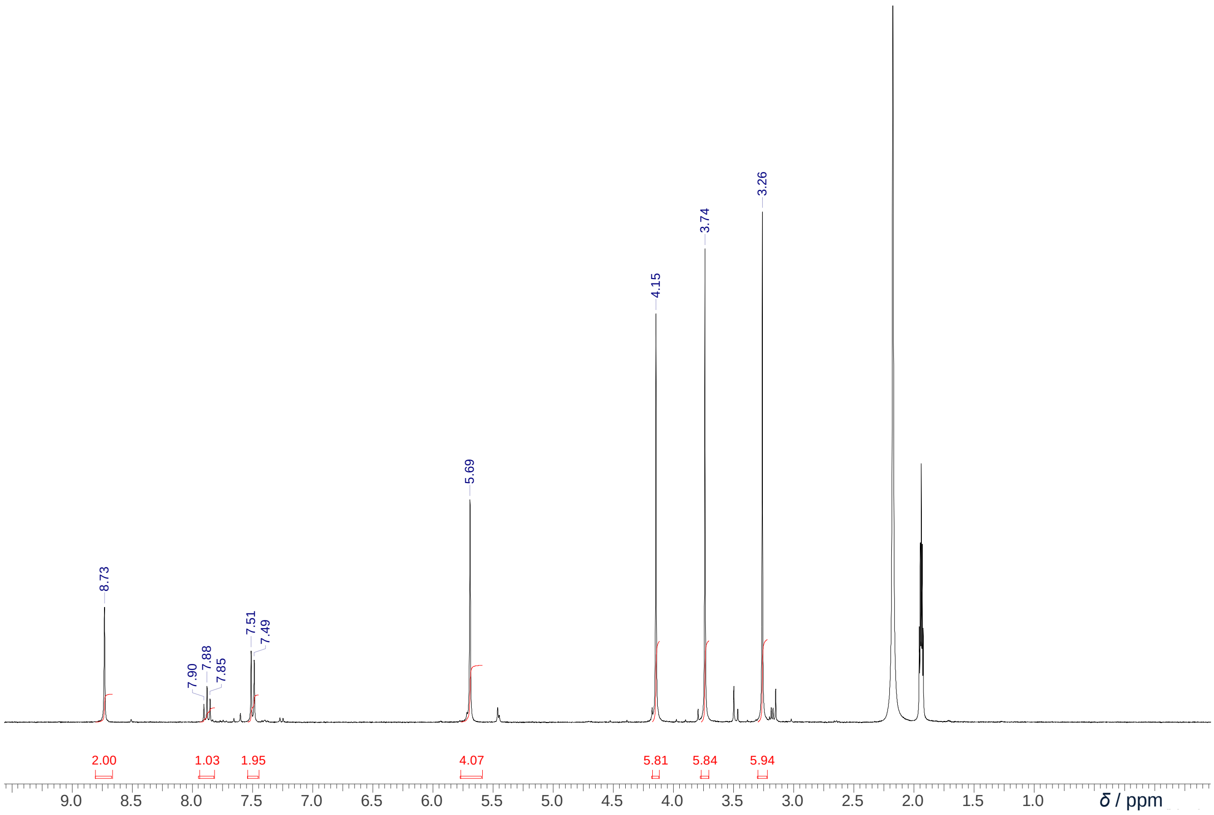


Figure S17: $^{1}$H-NMR (300 MHz, $CH_3CN$) of the ruthenium reaction.

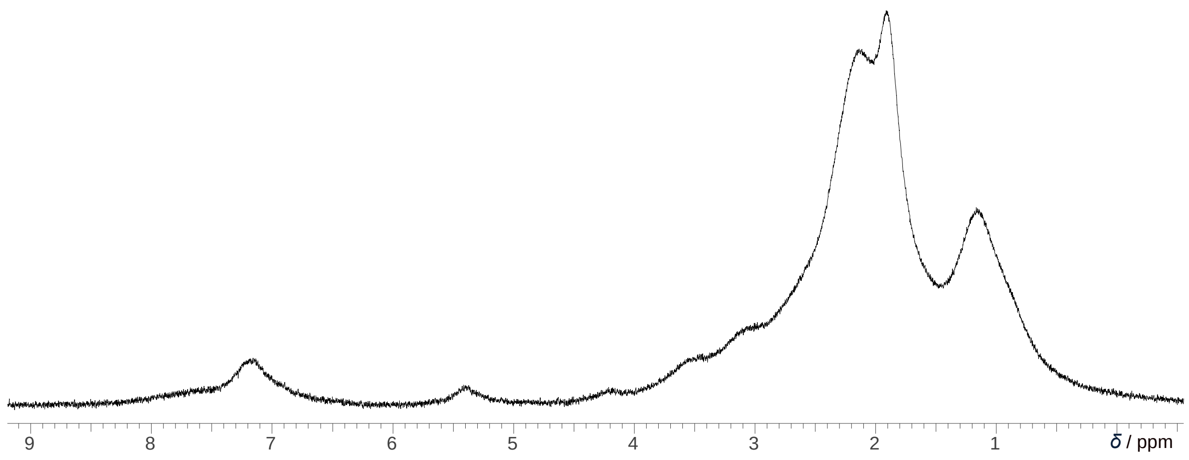


Figure S18: $^{1}$H-NMR (300 MHz, $CH_3CN$) of the iron(II) NHC complex.

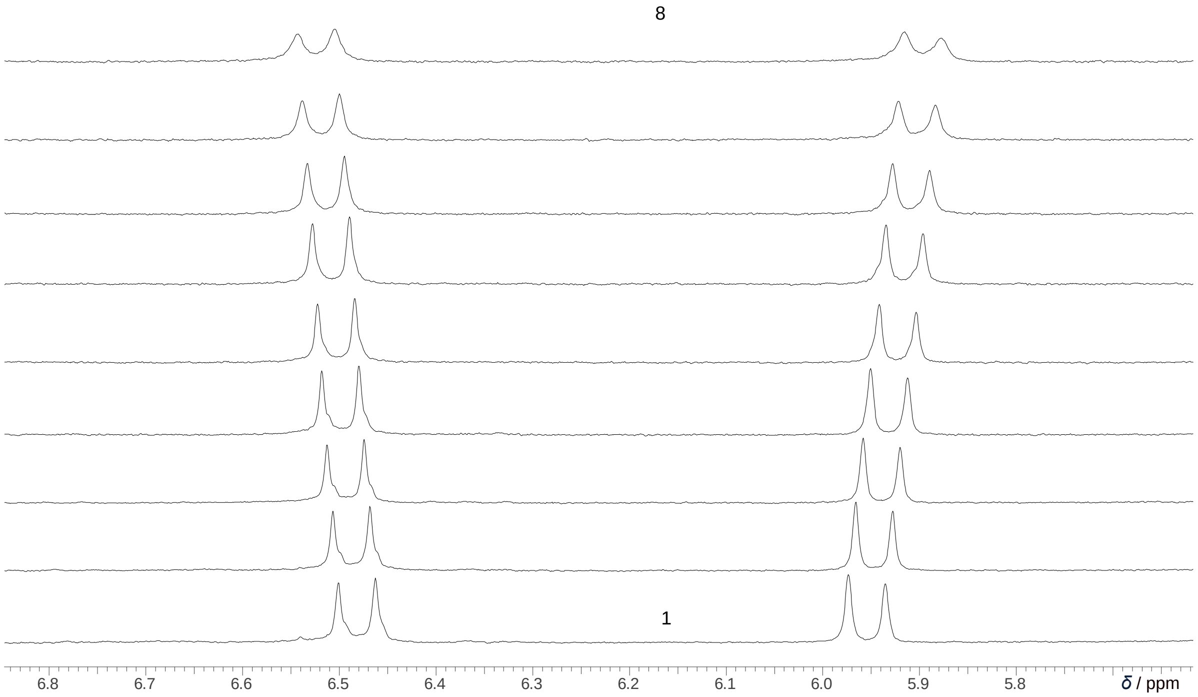


Figure S19: Variable temperature $^{1}$H-NMR (300 MHz, DMSO) of **6** from 25 °C (1) to 100 °C (8)

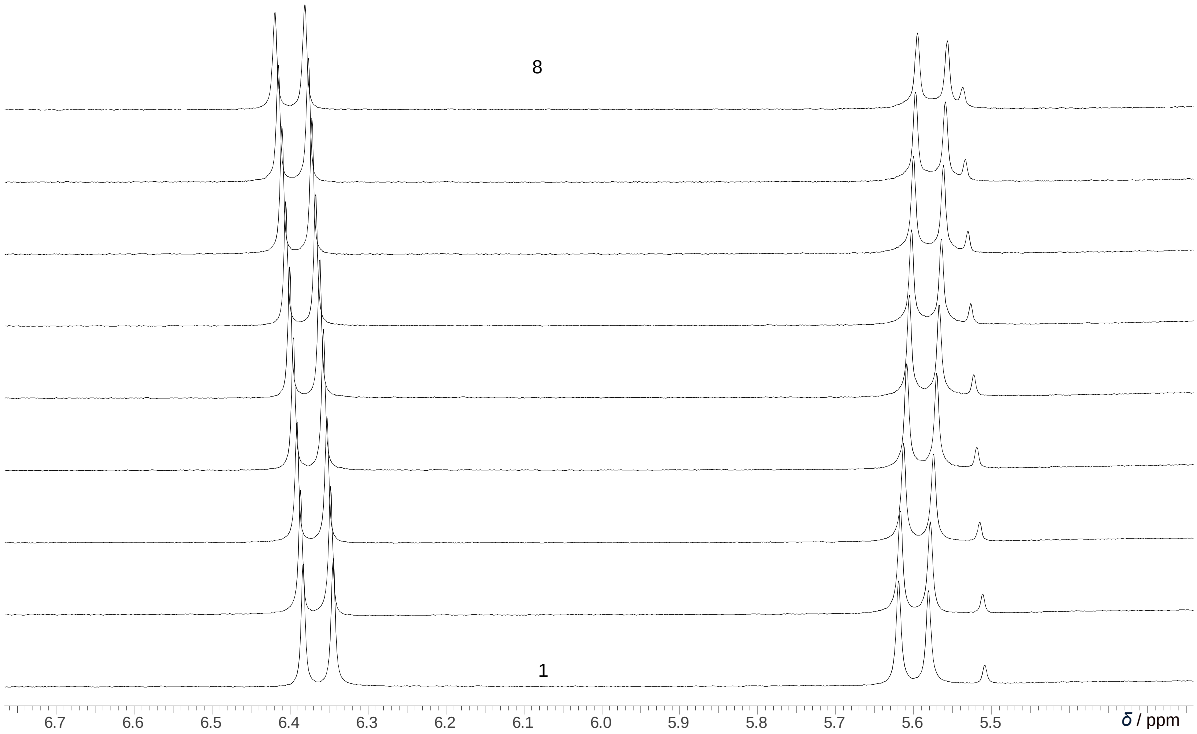


Figure S20: Variable temperature $^{1}$H-NMR (300 MHz, DMSO) of **7** from 25 °C (1) to 100 °C (8)

Table S1: Crystal data and details on structure refinement for **7** · $CH_3CN$.

| | |
|---|---|
| CSD deposition number | 2157149 |
| Internal identification code | bn00083 |
| Empirical formula | $C_{25}H_{28}ClF_6N_{10}O_4PPt$ |
| Formula weight | 908.08 |
| Temperature / K | 120 |
| Crystal system | triclinic |
| Space group | $P\bar{1}$ |
| $a$/Å | 10.430(1) |
| $b$/Å | 12.026(1) |
| $c$/Å | 13.200(1) |
| α/° | 81.610(2) |
| β/° | 88.365(2) |
| γ/° | 69.819(2) |
| Cell volume / $Å^3$ | 1537.1(2) |
| Z | 2 |
| Calcd. density $\rho_{calc}$ / $g/cm^3$ | 1.962 |
| Absorption coefficient μ / $mm^{-1}$ | 4.792 |
| $F$(000) | 888.0 |
| Crystal size / $mm^3$ | 0.29 × 0.21 × 0.17 |
| Crystal shape and color | colorless prism |
| Radiation | Mo-Kα (λ = 0.71073) |
| 2Θ range for data collection / ° | 3.646 to 67.042 |
| Index ranges | $-15 \leq h \leq 16$, $-16 \leq k \leq 18$, $-19 \leq l \leq 20$ |
| Reflections collected | 26731 |
| Independent reflections | 10968 [$R_{int}$ = 0.0289, $R_{sigma}$ = 0.0353] |
| Completeness of dataset | 100% |
| Data / restraints / parameters | 10968 / 0 / 440 |
| Goodness of fit on $F^2$ | 1.038 |
| Final $R$ indexes [$I$>=2σ($I$)] | $R_1$ = 0.0236, w$R_2$ = 0.0509 |
| Final $R$ indexes [all data] | $R_1$ = 0.0279, w$R_2$ = 0.0524 |
| Largest diff. peak and hole / e $Å^{-3}$ | 2.29 and –2.30 |

Table S2: Fractional Atomic Coordinates (×10[4]) and Equivalent Isotropic Displacement Parameters (Å[2]×10[3]) for **7** · $CH_3CN$. $U_{eq}$ is defined as 1/3 of the trace of the orthogonalised $U_{ij}$ tensor.

| Atom | *x* | *y* | *z* |
|---|---|---|---|
| Pt1 | 4790.5(2) | 3819.4(2) | 2730.2(2) |
| Cl1 | 6988.2(5) | 3716.0(5) | 2348.7(4) |
| O1 | 7398.5(18) | -1550.6(14) | 7067.0(13) |
| O2 | 3577.0(17) | 1795.7(15) | 6833.3(12) |
| O3 | 1446(2) | 9681.1(16) | -937.0(15) |
| O4 | 471.7(17) | 6217.7(15) | -447.0(12) |
| N1 | 2806.6(17) | 3960.2(14) | 3063.2(12) |
| N2 | 4634.2(17) | 2564.5(15) | 4762.4(13) |
| N3 | 6412.8(17) | 1376.1(15) | 4095.5(13) |
| N4 | 7081.2(18) | -261.8(15) | 5576.1(14) |
| N5 | 5465.7(19) | 84.7(16) | 6917.1(13) |
| N6 | 2928.1(18) | 5209.9(15) | 1030.8(13) |
| N7 | 4116.9(17) | 6294.6(16) | 1351.2(13) |
| N8 | 2823.9(19) | 8150.3(16) | 188.6(14) |
| N9 | 955.5(19) | 7958.1(17) | -680.5(14) |
| C1 | 5392.3(19) | 2474.8(18) | 3922.1(15) |
| C2 | 3975(2) | 5198.5(18) | 1606.4(15) |
| C3 | 3471(2) | 3653.1(18) | 4873.4(15) |
| C4 | 2413(2) | 3867.5(17) | 4047.6(15) |
| C5 | 1090(2) | 3948.2(18) | 4295.9(16) |
| C6 | 135(2) | 4140.7(19) | 3526.2(17) |
| C7 | 535(2) | 4249.3(18) | 2518.2(17) |
| C8 | 1881(2) | 4150.9(17) | 2305.7(15) |
| C9 | 2353(2) | 4244.4(19) | 1217.0(16) |
| C10 | 6277(2) | 816.6(18) | 5059.2(15) |
| C11 | 5139(2) | 1545.3(18) | 5474.4(15) |
| C12 | 6701(2) | -632.4(18) | 6551.1(17) |
| C13 | 4629(2) | 1210.7(18) | 6445.5(15) |
| C14 | 7452(2) | 910(2) | 3342.8(17) |
| C15 | 8463(2) | -931(2) | 5258(2) |
| C16 | 5092(3) | -397(2) | 7926.2(17) |
| C17 | 3115(2) | 6987.7(18) | 638.2(15) |
| C18 | 2364(2) | 6315.1(18) | 432.8(15) |
| C19 | 1721(2) | 8661(2) | -502.8(17) |
| C20 | 1209(2) | 6765.6(19) | -253.3(15) |
| C21 | 5063(2) | 6663(2) | 1915.8(18) |
| C22 | 3527(3) | 8934(2) | 464(2) |
| C23 | -182(2) | 8500(2) | -1433.4(19) |
| P1 | 1692.4(6) | 7596.1(6) | 3674.3(5) |
| F1 | 3183.3(17) | 6701.5(15) | 4044.9(13) |
| F2 | 1811.2(17) | 6976.5(17) | 2669.2(13) |
| F3 | 1029(2) | 6684.4(18) | 4263.8(16) |
| F4 | 1570.8(16) | 8218.4(15) | 4682.8(12) |
| F5 | 2371.4(18) | 8514.2(15) | 3087.4(13) |
| F6 | 213.3(16) | 8512.2(18) | 3301.7(14) |
| N10 | 2946(2) | 2013(2) | -258.2(17) |
| C24 | 2115(2) | 2216(2) | -868.1(18) |
| C25 | 1055(3) | 2463(2) | -1649.6(19) |

Table S3: Anisotropic Displacement Parameters (Å$^2$×10$^3$) for **7** · $CH_3CN$.

| **Atom** | **$U_{11}$** | **$U_{22}$** | **$U_{33}$** | **$U_{23}$** | **$U_{13}$** | **$U_{12}$** |
|---|---|---|---|---|---|---|
| Pt1 | 13.50(3) | 15.35(4) | 12.34(3) | 1.06(2) | 0.30(2) | -5.04(3) |
| Cl1 | 15.6(2) | 30.0(3) | 26.7(3) | 3.0(2) | 4.11(19) | -7.5(2) |
| O1 | 29.1(8) | 15.3(7) | 25.1(8) | 4.1(6) | -5.6(7) | -4.9(6) |
| O2 | 23.1(8) | 24.6(8) | 19.3(7) | 1.4(6) | 4.8(6) | -4.5(6) |
| O3 | 38.1(10) | 22.5(8) | 36.5(10) | 12.0(7) | -11.9(8) | -12.6(8) |
| O4 | 22.8(7) | 26.6(8) | 18.7(7) | 0.2(6) | -4.5(6) | -12.3(6) |
| N1 | 14.2(7) | 8.4(6) | 12.5(7) | 1.2(5) | -1.3(6) | -2.7(5) |
| N2 | 14.9(7) | 13.3(7) | 14.0(7) | 0.4(6) | 0.3(6) | -3.3(6) |
| N3 | 12.6(7) | 15.5(7) | 14.6(7) | -1.0(6) | -0.5(6) | -4.0(6) |
| N4 | 16.9(8) | 13.1(7) | 17.6(8) | 0.0(6) | -1.9(6) | -3.6(6) |
| N5 | 21.8(8) | 16.2(8) | 14.8(8) | 1.3(6) | -2.5(6) | -8.4(7) |
| N6 | 18.5(8) | 14.1(7) | 11.9(7) | 0.9(6) | -1.3(6) | -7.1(6) |
| N7 | 15.7(7) | 16.7(8) | 14.5(7) | 1.6(6) | -0.6(6) | -8.8(6) |
| N8 | 22.9(9) | 15.9(8) | 19.5(8) | 5.1(6) | -2.8(7) | -9.7(7) |
| N9 | 19.1(8) | 18.4(8) | 16.3(8) | 2.9(6) | -2.4(6) | -5.5(7) |
| C1 | 13.6(8) | 15.4(8) | 12.8(8) | -0.2(7) | -1.6(6) | -4.9(7) |
| C2 | 16.2(8) | 15.6(8) | 12.4(8) | 0.3(7) | 0.2(7) | -6.8(7) |
| C3 | 16.7(8) | 13.3(8) | 13.7(8) | -1.3(7) | 1.4(7) | -2.8(7) |
| C4 | 15.6(8) | 10.3(8) | 15.3(8) | -0.3(6) | 0.9(7) | -3.3(7) |
| C5 | 17.9(9) | 14.9(9) | 17.7(9) | -1.6(7) | 3.3(7) | -5.7(7) |
| C6 | 15.9(9) | 18.4(9) | 24.9(10) | -1.4(8) | 1.6(8) | -6.8(7) |
| C7 | 16.9(9) | 15.3(9) | 21.1(10) | -0.3(7) | -3.0(7) | -6.6(7) |
| C8 | 17.7(8) | 11.1(8) | 16.9(9) | 0.4(7) | -0.8(7) | -6.0(7) |
| C9 | 22.1(9) | 16.2(9) | 15.2(9) | -0.9(7) | -1.6(7) | -10.1(8) |
| C10 | 14.7(8) | 13.7(8) | 14.9(8) | 0.0(7) | -2.1(7) | -5.7(7) |
| C11 | 14.6(8) | 14.3(8) | 14.4(8) | -0.1(7) | -1.1(7) | -4.6(7) |
| C12 | 20.3(9) | 14.0(9) | 19.9(9) | -0.8(7) | -3.9(7) | -7.6(7) |
| C13 | 19.4(9) | 16.6(9) | 14.2(8) | 0.5(7) | -2.1(7) | -8.5(7) |
| C14 | 18.1(9) | 23.7(10) | 18.5(9) | -4.5(8) | 5.1(7) | -3.5(8) |
| C15 | 18.7(10) | 17.0(10) | 32.9(12) | 2.8(9) | 2.3(9) | 0.0(8) |
| C16 | 32.3(12) | 22.6(10) | 14.7(9) | 3.5(8) | -1.3(8) | -11.2(9) |
| C17 | 16.5(8) | 16.9(9) | 13.4(8) | 1.6(7) | 0.1(7) | -7.1(7) |
| C18 | 19.0(9) | 16.5(9) | 11.5(8) | 0.2(7) | -0.4(7) | -7.7(7) |
| C19 | 23.7(10) | 20.6(10) | 19.1(10) | 3.4(8) | -1.9(8) | -7.5(8) |
| C20 | 17.9(9) | 19.1(9) | 11.8(8) | -0.1(7) | 0.7(7) | -6.6(7) |
| C21 | 23.1(10) | 24.2(10) | 22.4(10) | 3.7(8) | -6.8(8) | -14.9(9) |
| C22 | 33.1(13) | 20.3(11) | 40.9(14) | 7.5(10) | -11.1(11) | -16.5(10) |
| C23 | 22.3(10) | 29.5(12) | 25.7(11) | 8.1(9) | -8.9(9) | -7.2(9) |
| P1 | 19.0(2) | 24.4(3) | 23.6(3) | -8.8(2) | 4.3(2) | -10.9(2) |
| F1 | 27.9(8) | 34.6(9) | 40.1(9) | 1.0(7) | -0.3(7) | -2.2(7) |
| F2 | 30.2(8) | 59.2(11) | 40.0(9) | -33.6(9) | 10.6(7) | -15.9(8) |
| F3 | 60.3(12) | 50.1(11) | 62.1(12) | -23.5(10) | 32.4(10) | -43.4(10) |
| F4 | 33.5(8) | 42.1(9) | 29.0(8) | -17.0(7) | 0.7(6) | -16.3(7) |
| F5 | 44.9(10) | 34.1(9) | 39.1(9) | 3.3(7) | 6.0(7) | -21.6(8) |
| F6 | 22.8(7) | 62.0(12) | 41.5(10) | -26.1(9) | -5.1(7) | 2.3(8) |
| N10 | 29.2(10) | 30.6(11) | 29.5(11) | -2.9(9) | 1.6(8) | -13.7(9) |
| C24 | 24.0(10) | 19.3(10) | 23.7(10) | -2.8(8) | 6.0(8) | -8.7(8) |
| C25 | 25.2(11) | 28.4(12) | 23.9(11) | -2.0(9) | 0.8(9) | -6.5(9) |

Table S4: Bond lengths for **7** · $CH_3CN$.

| Atom | Atom | Length/Å | Atom | Atom | Length/Å |
|---|---|---|---|---|---|
| Pt1 | Cl1 | 2.2976(6) | N7 | C17 | 1.378(3) |
| Pt1 | N1 | 2.0571(16) | N7 | C21 | 1.471(3) |
| Pt1 | C1 | 2.024(2) | N8 | C17 | 1.369(3) |
| Pt1 | C2 | 2.014(2) | N8 | C19 | 1.393(3) |
| O1 | C12 | 1.211(3) | N8 | C22 | 1.467(3) |
| O2 | C13 | 1.226(3) | N9 | C19 | 1.394(3) |
| O3 | C19 | 1.215(3) | N9 | C20 | 1.398(3) |
| O4 | C20 | 1.225(3) | N9 | C23 | 1.471(3) |
| N1 | C4 | 1.352(2) | C3 | C4 | 1.505(3) |
| N1 | C8 | 1.349(3) | C4 | C5 | 1.382(3) |
| N2 | C1 | 1.339(2) | C5 | C6 | 1.381(3) |
| N2 | C3 | 1.469(3) | C6 | C7 | 1.385(3) |
| N2 | C11 | 1.382(3) | C7 | C8 | 1.391(3) |
| N3 | C1 | 1.374(3) | C8 | C9 | 1.507(3) |
| N3 | C10 | 1.377(3) | C10 | C11 | 1.364(3) |
| N3 | C14 | 1.469(3) | C11 | C13 | 1.431(3) |
| N4 | C10 | 1.369(3) | C17 | C18 | 1.362(3) |
| N4 | C12 | 1.392(3) | C18 | C20 | 1.426(3) |
| N4 | C15 | 1.472(3) | P1 | F1 | 1.5970(17) |
| N5 | C12 | 1.399(3) | P1 | F2 | 1.5957(16) |
| N5 | C13 | 1.397(3) | P1 | F3 | 1.5910(18) |
| N5 | C16 | 1.470(3) | P1 | F4 | 1.6012(15) |
| N6 | C2 | 1.343(3) | P1 | F5 | 1.6060(17) |
| N6 | C9 | 1.468(3) | P1 | F6 | 1.5963(18) |
| N6 | C18 | 1.384(3) | N10 | C24 | 1.139(3) |
| N7 | C2 | 1.369(3) | C24 | C25 | 1.457(3) |

Table S5: Bond Angles for **7** · $CH_3CN$.

| Atom | Atom | Atom | Angle/° | Atom | Atom | Atom |
|---|---|---|---|---|---|---|
| N1 | Pt1 | Cl1 | 178.50(5) | C6 | C7 | C8 |
| C1 | Pt1 | Cl1 | 93.38(6) | N1 | C8 | C7 |
| C1 | Pt1 | N1 | 87.80(7) | N1 | C8 | C9 |
| C2 | Pt1 | Cl1 | 92.99(6) | C7 | C8 | C9 |
| C2 | Pt1 | N1 | 85.82(7) | N6 | C9 | C8 |
| C2 | Pt1 | C1 | 173.46(8) | N4 | C10 | N3 |
| C4 | N1 | Pt1 | 120.24(13) | C11 | C10 | N3 |
| C8 | N1 | Pt1 | 120.64(13) | C11 | C10 | N4 |
| C8 | N1 | C4 | 119.12(17) | N2 | C11 | C13 |
| C1 | N2 | C3 | 121.68(17) | C10 | C11 | N2 |
| C1 | N2 | C11 | 110.88(16) | C10 | C11 | C13 |
| C11 | N2 | C3 | 127.39(16) | O1 | C12 | N4 |
| C1 | N3 | C10 | 109.02(16) | O1 | C12 | N5 |
| C1 | N3 | C14 | 123.62(17) | N4 | C12 | N5 |
| C10 | N3 | C14 | 127.35(17) | O2 | C13 | N5 |
| C10 | N4 | C12 | 118.40(17) | O2 | C13 | C11 |
| C10 | N4 | C15 | 123.29(18) | N5 | C13 | C11 |
| C12 | N4 | C15 | 117.05(18) | N8 | C17 | N7 |
| C12 | N5 | C16 | 114.73(18) | C18 | C17 | N7 |
| C13 | N5 | C12 | 126.32(18) | C18 | C17 | N8 |
| C13 | N5 | C16 | 118.85(18) | N6 | C18 | C20 |
| C2 | N6 | C9 | 121.18(17) | C17 | C18 | N6 |
| C2 | N6 | C18 | 110.31(17) | C17 | C18 | C20 |
| C18 | N6 | C9 | 127.34(17) | O3 | C19 | N8 |
| C2 | N7 | C17 | 108.94(16) | O3 | C19 | N9 |
| C2 | N7 | C21 | 121.95(17) | N8 | C19 | N9 |
| C17 | N7 | C21 | 128.33(18) | O4 | C20 | N9 |
| C17 | N8 | C19 | 118.79(18) | O4 | C20 | C18 |
| C17 | N8 | C22 | 123.61(19) | N9 | C20 | C18 |
| C19 | N8 | C22 | 117.34(18) | F1 | P1 | F4 |
| C19 | N9 | C20 | 126.24(18) | F1 | P1 | F5 |
| C19 | N9 | C23 | 116.88(19) | F2 | P1 | F1 |
| C20 | N9 | C23 | 116.78(18) | F2 | P1 | F4 |
| N2 | C1 | Pt1 | 118.33(14) | F2 | P1 | F5 |
| N2 | C1 | N3 | 106.17(17) | F2 | P1 | F6 |
| N3 | C1 | Pt1 | 135.39(14) | F3 | P1 | F1 |
| N6 | C2 | Pt1 | 119.40(14) | F3 | P1 | F2 |
| N6 | C2 | N7 | 106.53(17) | F3 | P1 | F4 |
| N7 | C2 | Pt1 | 133.34(15) | F3 | P1 | F5 |
| N2 | C3 | C4 | 109.59(16) | F3 | P1 | F6 |
| N1 | C4 | C3 | 117.74(17) | F4 | P1 | F5 |
| N1 | C4 | C5 | 121.59(19) | F6 | P1 | F1 |
| C5 | C4 | C3 | 120.66(18) | F6 | P1 | F4 |
| C6 | C5 | C4 | 119.76(19) | F6 | P1 | F5 |
| C5 | C6 | C7 | 118.60(19) | N10 | C24 | C25 |

Table S6: Hydrogen Atom Coordinates (Å×$10^4$) and Isotropic Displacement Parameters (Å$^2$×$10^3$) for **7** · $CH_3CN$.

| **Atom** | ***x*** | ***y*** | ***z*** | **U(eq)** |
|---|---|---|---|---|
| H3A | 3786.44 | 4346.99 | 4820.38 | 18 |
| H3B | 3067.66 | 3561.99 | 5554.87 | 18 |
| H5 | 839.05 | 3871.63 | 4993.22 | 20 |
| H6 | -777.62 | 4197.33 | 3685.22 | 24 |
| H7 | -104.84 | 4390.12 | 1974.99 | 21 |
| H9A | 3055.17 | 3476.72 | 1101.23 | 20 |
| H9B | 1573.69 | 4416.76 | 737.28 | 20 |
| H14A | 7178.3 | 1403.96 | 2673.08 | 32 |
| H14B | 7539.21 | 82.05 | 3291.34 | 32 |
| H14C | 8331.6 | 931.75 | 3562 | 32 |
| H15A | 8397.8 | -1359.1 | 4694.57 | 38 |
| H15B | 8967 | -1507.79 | 5837.87 | 38 |
| H15C | 8944.6 | -372.37 | 5028.12 | 38 |
| H16A | 5829.76 | -540.39 | 8425.56 | 35 |
| H16B | 4947.45 | -1151.15 | 7881.12 | 35 |
| H16C | 4249.61 | 181.05 | 8144.54 | 35 |
| H21A | 4538.64 | 7306.79 | 2299.39 | 33 |
| H21B | 5647.76 | 6950.98 | 1431.05 | 33 |
| H21C | 5631.12 | 5978.13 | 2393.13 | 33 |
| H22A | 3393.14 | 9019.81 | 1190.29 | 46 |
| H22B | 3152.51 | 9723.16 | 46.33 | 46 |
| H22C | 4505.82 | 8584.04 | 339.31 | 46 |
| H23A | 96.57 | 8198.61 | -2085.49 | 41 |
| H23B | -425.86 | 9372.32 | -1535.96 | 41 |
| H23C | -972.73 | 8289.27 | -1180.45 | 41 |
| H25A | 1457.65 | 2482.89 | -2329.12 | 40 |
| H25B | 347.38 | 3239.41 | -1599.64 | 40 |
| H25C | 647.78 | 1833.94 | -1545.9 | 40 |
